# Clonal origins and parallel evolution of regionally synchronous colorectal adenoma and carcinoma

**DOI:** 10.18632/oncotarget.4834

**Published:** 2015-08-07

**Authors:** Tae-Min Kim, Chang Hyeok An, Je-Keun Rhee, Seung-Hyun Jung, Sung Hak Lee, In-Pyo Baek, Min Sung Kim, Sug Hyung Lee, Yeun-Jun Chung

**Affiliations:** ^1^ Departments of Medical Informatics, College of Medicine, The Catholic University of Korea, Seoul, South Korea; ^2^ Departments of Cancer Evolution Research Center, College of Medicine, The Catholic University of Korea, Seoul, South Korea; ^3^ Departments of Surgery, College of Medicine, The Catholic University of Korea, Seoul, South Korea; ^4^ Departments of Integrated Research Center for Genome Polymorphism, College of Medicine, The Catholic University of Korea, Seoul, South Korea; ^5^ Departments of Hospital Pathology, College of Medicine, The Catholic University of Korea, Seoul, South Korea; ^6^ Departments of Pathology, College of Medicine, The Catholic University of Korea, Seoul, South Korea; ^7^ Departments of Microbiology, College of Medicine, The Catholic University of Korea, Seoul, South Korea

**Keywords:** colorectal cancer, carcinogenesis, exome sequencing, mutations, evolution

## Abstract

Although the colorectal adenoma-to-carcinoma sequence represents a classical cancer progression model, the evolution of the mutational landscape underlying this model is not fully understood. In this study, we analyzed eight synchronous pairs of colorectal high-grade adenomas and carcinomas, four microsatellite-unstable (MSU) and four -stable (MSS) pairs, using whole-exome sequencing. In the MSU adenoma-carcinoma pairs, we observed no subclonal mutations in adenomas that became fixed in paired carcinomas, suggesting a ‘parallel’ evolution of synchronous adenoma-to-carcinoma, rather than a ‘stepwise’ evolution. The abundance of indel (in MSU and MSS pairs) and microsatellite instability (in MSU pairs) was noted in the later adenoma- or carcinoma-specific mutations, indicating that the mutational processes and functional constraints operative in early and late colorectal carcinogenesis are different. All MSU cases exhibited clonal, truncating mutations in *ACVR2A*, *TGFBR2*, and DNA mismatch repair genes, but none were present in *APC* or *KRAS*. In three MSS pairs, both *APC* and *KRAS* mutations were identified as both early and clonal events, often accompanying clonal copy number changes. An MSS case uniquely exhibited clonal *ERBB2* amplification, followed by *APC* and *TP53* mutations as carcinoma-specific events. Along with the previously unrecognized clonal origins of synchronous colorectal adenoma-carcinoma pairs, our study revealed that the preferred sequence of mutational events during colorectal carcinogenesis can be context-dependent.

## INTRODUCTION

Colorectal cancer (CRC) is the third most common human cancer worldwide and is also a major contributor to cancer-related mortality [1]. Although early detection and prevention have reduced overall CRC-related risks [2], the advanced disease states remain incurable, with few therapeutic options. Although large-scale efforts such as the Cancer Genome Atlas consortium [3, 4] have led to a previously unrecognized molecular understanding of CRC carcinogenesis, the evolutionary scope and insights that can be obtained from the genomic snapshots of the fully developed tumors in these cohorts may be limited.

For CRC carcinogenesis, progression from cellular dysplasia to malignancy has been well studied [5]. This classical cancer evolution model describes CRC carcinogenesis as a series of well-defined clinical stages accompanying a stepwise accumulation of somatic oncogenic mutations that are known to contribute to the malignant progression [5]. According to this model, it is generally accepted that the malignant lesion originates from a pre-existing adenoma. Although adenoma tissues are typically destroyed during malignant progression, residual adenomas often continue to exist with the carcinoma lesions. Such synchronous adenomas and carcinomas present an unusual setting where the temporal evolution of the mutational landscape during the adenoma-to-carcinoma progression in a given individual can be investigated by simultaneous genomic profiling of benign and malignant lesions from a single individual [6–8].

In addition, the use of advanced genomic profiling techniques such as next-generation sequencing has enabled cancer evolution studies. For example, whole-genome sequencing-based inference of subclonal architectures using the burdens of mutant alleles has revealed the evolution of subclones during the progression of cancers [9, 10]. Given that a colorectal adenoma-to-carcinoma transition is an evolutionary process and is encrypted within the tumor genomes, evolutionary perspectives in terms of genome- or exome-wide mutational abundance and their clonal distribution may provide valuable insights into the mechanism of CRC progression, with potential clinical benefits. Specifically, the comparison of genomic footprints between synchronous adenoma-*vs*.-carcinoma lesions might aid in the (i) inference of the evolutionary history of synchronous lesions during CRC carcinogenesis, (ii) recovery of the mutational profiles of ancestral cancer genomes, and (iii) identification of the temporal sequences in the acquisition of somatic mutations.

Here, we performed whole exome-based mutational analyses for eight synchronous pairs of colorectal adenomas and carcinomas. Adenoma tissues were separated from accompanying carcinomas using microdissection and subjected to whole-exome sequencing (WES). Given the unique molecular pathogenic features between microsatellite-stable (MSS) and microsatellite-unstable (MSU) CRCs [11], adenoma-carcinoma pairs from both MSS and MSU CRCs were explored in this study. We show that the mutational architecture supports a parallel evolution, with clonal origins of synchronous benign and malignant lesions instead of the traditional adenoma-to-carcinoma sequence. We also observed that sequential acquisition of key somatic mutations during colorectal malignant transformation is largely consistent with previous reports (e.g., the early, clonal appearance of *APC* and *KRAS* mutations in microsatellite-stable CRC), but this might often be context-dependent.

## RESULTS

### Whole-exome sequencing and somatic variants

**Figure 1:**
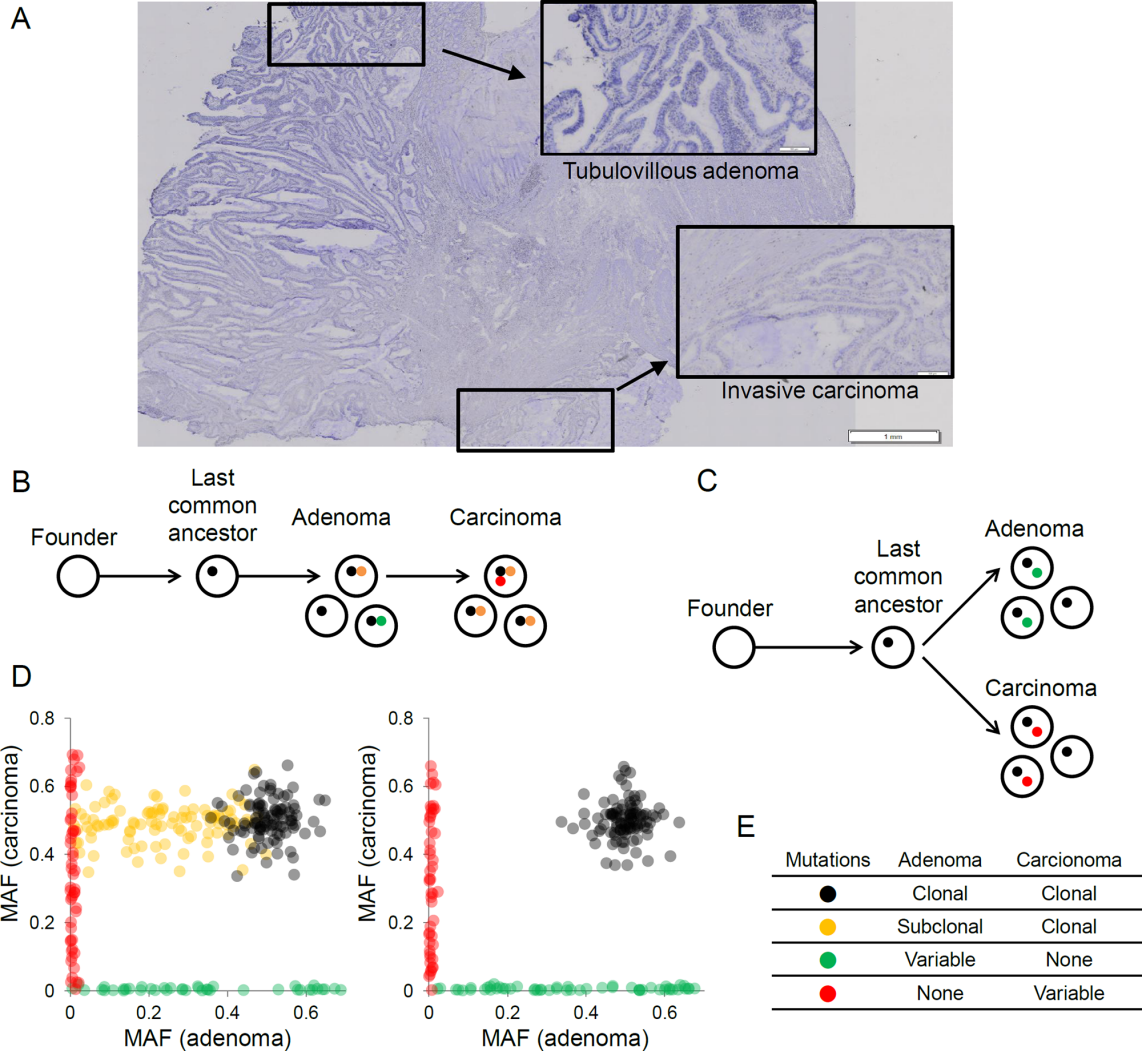
The evolutionary relationships of synchronous adenoma-carcinoma lesions inferred from the clonal architecture of somatic mutations **A.** Histology of synchronous adenoma and carcinoma in a representative case of colon cancer is shown. High-grade tubulovillous adenoma (inset) and invasive carcinoma (inset) lesions were microdissected and used for genomic analyses. **B.** A schematic of carcinoma arising from a clone in the preceding adenoma is shown. Representative mutations that occur during the adenoma-carcinoma progression are shown in the respective colors (stepwise evolution). **C.** A schematic of both adenoma and carcinoma arising from a single clone representing the last common ancestor (parallel evolution) is shown. **D.** The distribution of mutational allele frequencies (MAF) supporting stepwise evolution (left) and parallel evolution (right) is shown. **E.** The expected clonalities of mutations in adenoma and carcinoma lesions are shown for four mutational classes. Variable indicates that the mutations can be either clonal or subclonal.

To obtain mutational landscapes for synchronous colorectal adenomas and carcinomas, we performed WES for eight genome pairs of colorectal high-grade adenomas and carcinomas as well as for matched adjacent normal tissues. To exclude potential metachronous lesions with independent evolutionary origins, synchronous lesions were obtained in a histologically defined, single cancer mass and were carefully separated using microdissection (Figure 1A). Clinicopathologic information of the four MSS (MSS1-4) and four MSU (MSU1-4) cases is shown in Table 1. Three types of somatic variants, i.e., single nucleotide variants (SNVs), small insertions/deletions (indels), and microsatellite instability (MSI) events, were identified by comparing the paired-end WES data of tumor genomes with those of the matched normal controls. We obtained a total of 11,250 somatic variants, which are presented in Supplementary Table S1. We also identified alterations in somatic copy number using the sequencing read depth difference between the tumor and matched normal control exome sequencing data.

**Table 1:** Clinicopathologic parameters of eight CRC patients

Case	Age/sex	MSI status	Location	Diameter of primary cancer (cm)	Differentiation	T	N	M	TNM	Associated adenomas	Tumor cell content (%)
MSS1	M/69	MSS	Sigmoid	6.0	Poor	3	2	1	IVA	Tubular, high grade	>70
MSS2	M/61	MSS	Sigmoid	11 cm	Well	4	0	0	IIB	Tubulovillous, high grade	>70
MSS3	F/69	MSS	Transverse	8 cm	Well	2	0	0	I	Tubular, high grades	>70
MSS4	F/80	MSS	Ascending	4.5 cm	Moderate	3	0	0	IIA	Villous, high grade	>70
MSU1	M/68	MSI-H	Ascending	10 cm	Mucinous	3	0	0	IIA	Tubulovillous, high grade	>70
MSU2	M/66	MSI-H	Cecal	7.0 cm	Mucinous	3	0	0	IIA	Villous, high grade	>70
MSU3	F/51	MSI-H	Transverse	5.0 cm	Moderate	3	0	0	IIA	Tubulovillous, high grade	>70
MSU4	F/76	MSI-H	Ascending	9.5 cm	Moderate	3	2	0	IIB	Tubulovillous, high grade	>70

The eight cases are distinguished into four MSS (microsatellite stable) cases (MSS1-4) and four MSU (microsatellite unstable) cases (MSU1-4) with MSI-H (microsatellite instability-high) according to the MSI calls.

### Parallel-vs.-stepwise evolution of synchronous colorectal adenomas and carcinomas

To investigate the evolutionary relationship of synchronous adenoma and carcinoma lesions, we hypothesized two possible scenarios. The first is that a carcinoma arises from a clone among the cells in the preceding adenoma (stepwise evolution, Figure 1B). The second is that both the adenoma and carcinoma independently originate from a single, common progenitor cell or the last common ancestor (parallel evolution, Figure 1C). Clonal analyses of mutations from synchronous lesions that are based on the numbers and burdens of mutant alleles might distinguish mutations arising at different evolutionary stages, and these mutations might be used as markers to evaluate an appropriate evolutionary model [10]. Here, we propose that the somatic mutations in synchronous adenoma-carcinoma lesions can be distinguished into at least four classes. First, the mutations that have arisen early in cancer development (i.e., those acquired from the emergence of a founder cell and to the last common ancestor) will be clonal and commonly observed across the tumor regions (black in Figure 1). Next, the mutations that arise after the divergence of clones representing adenomas and carcinomas will appear lesion-specific (Figure 1, green and red, respectively). The presence of mutations that are clonal in the carcinoma but subclonal in the adenoma (orange in Figure 1) suggests that the malignant clone is selected among subclones in the preceding adenoma, thus supporting a stepwise evolution instead of a parallel evolution. The presence of these mutations will be a key in determining the appropriate evolutionary model. Two examples of mutant allele frequency (MAF)-based scatter plots corresponding to stepwise and parallel evolutions are illustrated in Figure 1D. The expected clonalities for these four classes of mutations are summarized in Figure 1E.

### Clonal analyses using mutant allele abundance in microsatellite-unstable genomes

**Figure 2:**
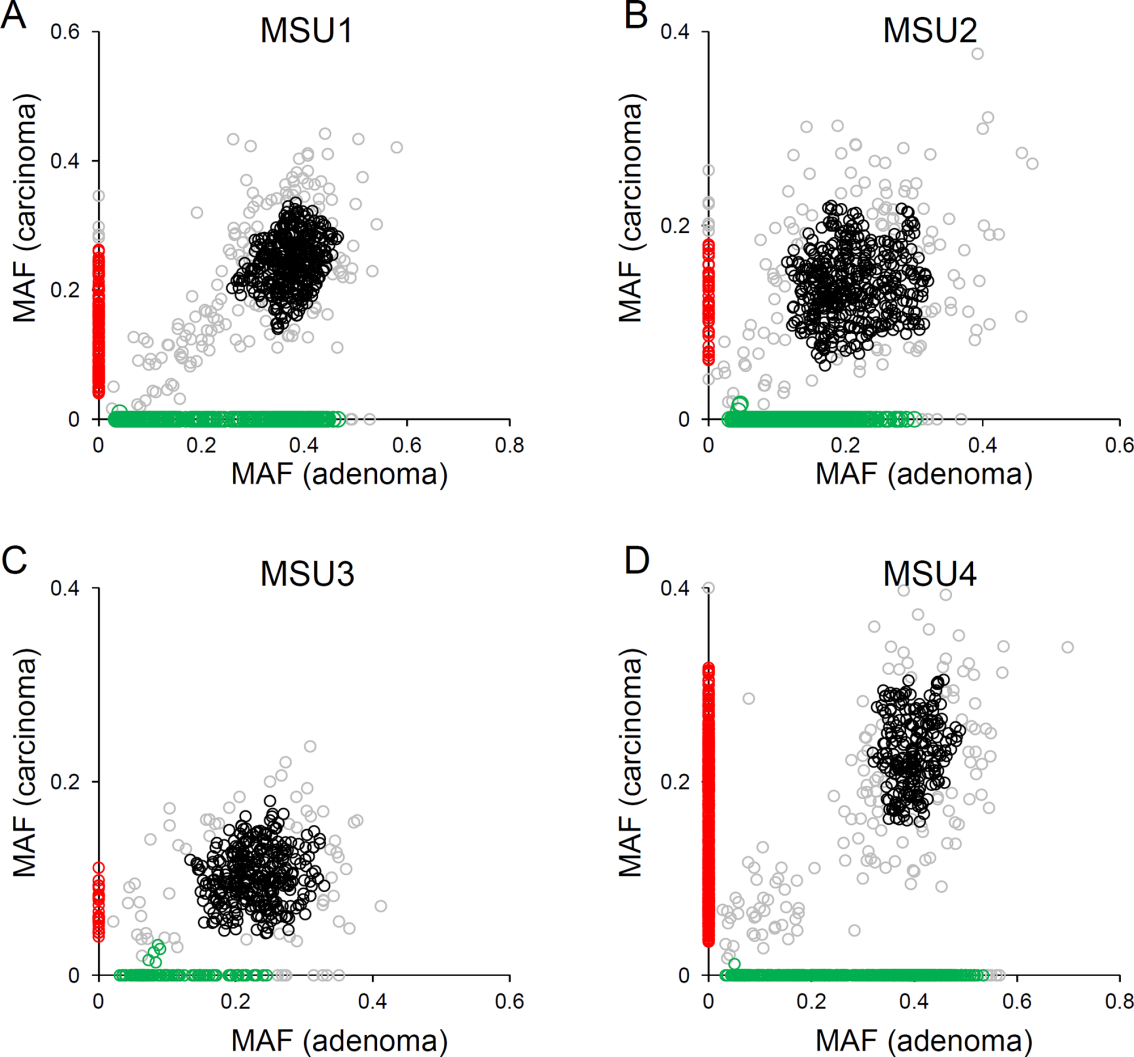
Clonal analyses using mutational abundance microsatellite-unstable genomes **A.** The distribution of MAF in adenoma (x-axis) and carcinoma (y-axis) is illustrated for MSU1. Four mutation clusters are distinguished with respective clones (black, clonal; green, adenoma-specific; red, carcinoma-specific). The mutations that do not belong to these three classes are shown in gray. **B–D.** Similar representations are shown for the other three MSU cases.

For mutation-based clonal analysis, we selected four MSU cases to ensure a sufficient number of somatic mutations are analyzed as evolutionary markers. Figure 2 shows the distribution of the MAF of somatic SNVs in four MSU cases. Unsupervised clustering defined four mutation clusters in each of the four MSU genomes: clonal (black), adenoma- and carcinoma-specific mutations (green and red respectively), and outliers (grey). Importantly, no distinct cluster representing the mutations that were subclonal in adenomas but clonal in carcinomas was observed in the four MSU genomes examined. This result is suggestive of parallel evolution of synchronous adenoma and carcinoma lesions, i.e., the emergence of subclones arising from a last common ancestor that already acquired a substantial number of somatic mutations (clonal) with independent outgrowth subsequently acquiring lesion-specific somatic mutations. In the four MSS genomes, we were unable to observe any conclusive evidence for parallel or stepwise evolution, since MSS genomes harbor fewer somatic mutations than MSU genomes (Supplementary Figure S1).

### Mutation signatures with respect to regional mutation categories

**Figure 3:**
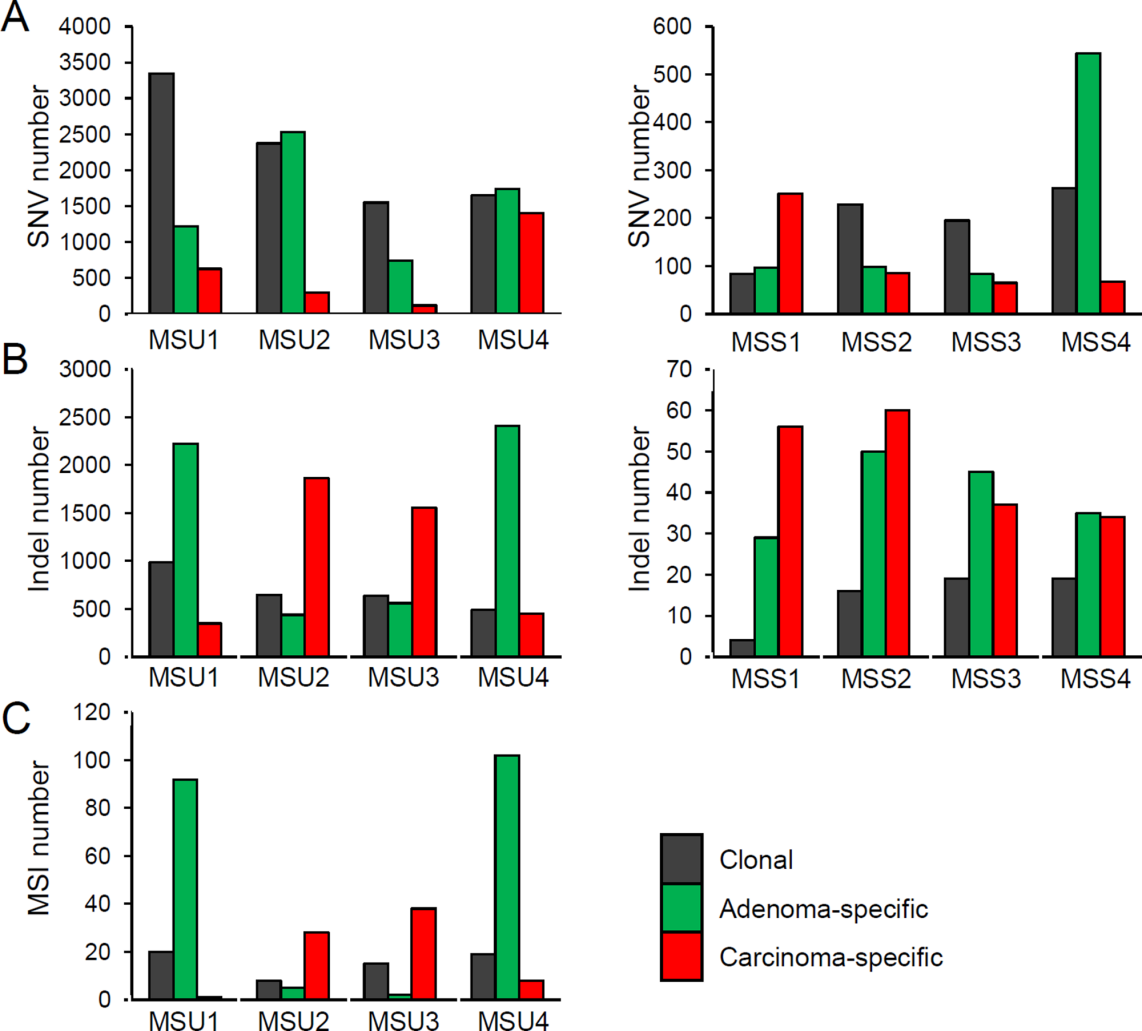
Abundance of somatic mutations **A.** The SNV abundance is shown for three regional classes (clonal as well as adenoma- and carcinoma-specific mutations, in the indicated colors). Four MSU and four MSS cases are shown in the left and right panels, respectively. **B.** The indel abundance is shown similarly. **C.** The MSI abundance is shown for four MSU cases.

According to the clonal origins of synchronous lesions, we categorized the somatic variants into ‘clonal’ (i.e., those commonly observed in both lesions) and those specific in adenoma and carcinoma (‘adenoma’ and ‘carcinoma’, respectively). Figure 3 shows the mutational abundance of these three regional mutational categories in the eight adenoma-carcinoma pairs, separately for MSU and MSS cases. First, clonal SNVs outnumbered the lesion-specific SNVs in half of the cases, but SNV abundance was variable within and across the cases (Figure 3A). To the contrary, the lesion-specific indels outnumbered clonal indels in both MSU and MSS genomes (Figure 3B). The lesion-specific prevalence of indels was consistently observed for high-confidence indels (e.g., those with MAF >40% or covered >50X, Supplementary Figure S2) as well as for independently called MSI events (Figure 3C), suggesting that this observation is not merely due to technical issues such as the sensitivity of indel calling. In addition, no substantial differences were observed in either functional annotations or mutation spectra of SNVs across the cases or the regional categories (Supplementary Figure S3).

### Mutational map of microsatellite-stable CRC genomes

**Figure 4:**
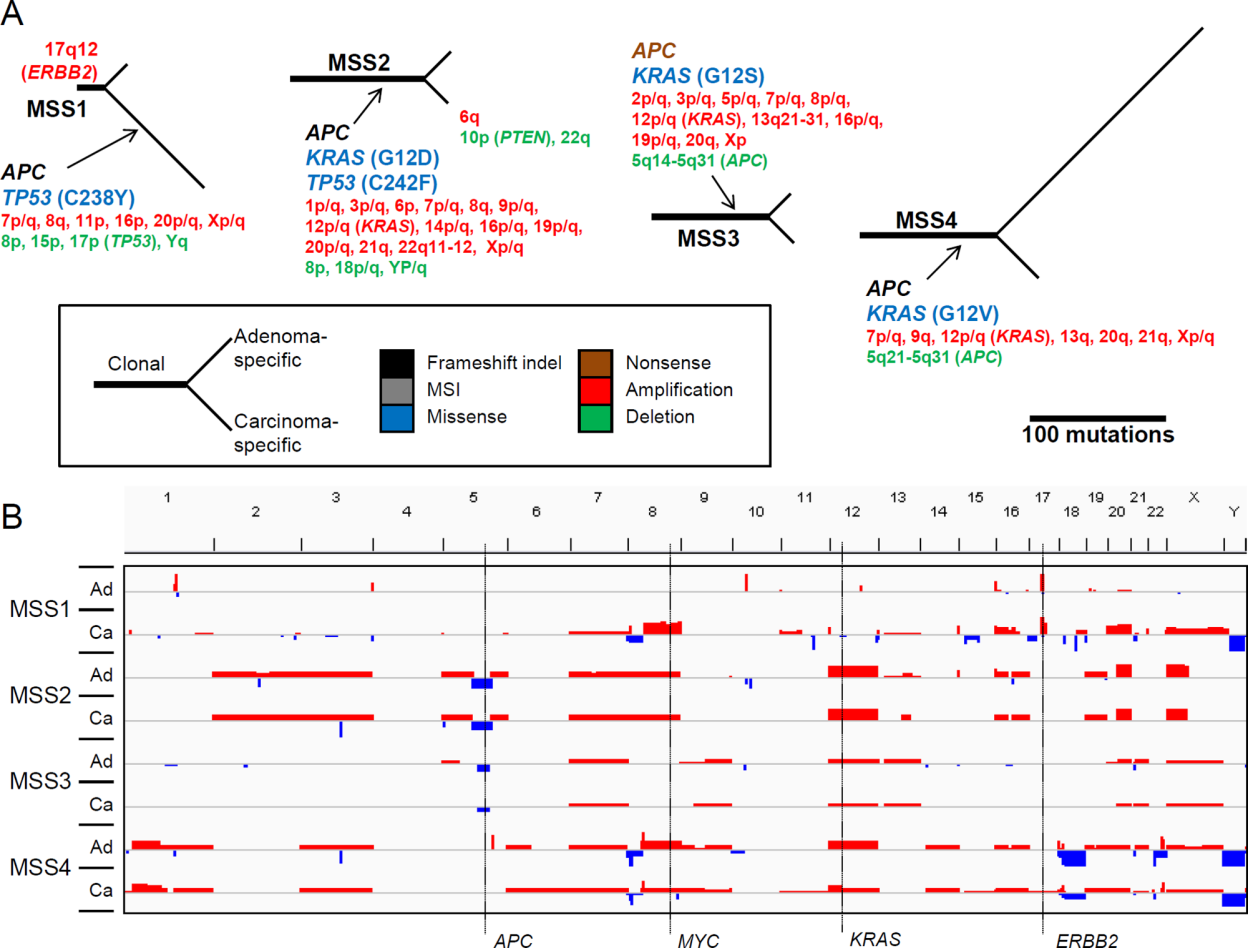
Mutational map and copy number heatmap of MSS genomes **A.** In the tree, the trunk and two branches represent the clonal mutations and adenoma-specific (upper branches) and carcinoma-specific (lower branches) mutations, respectively. The length of the branches is proportional to the number of somatic variants identified. The gene symbols represent the nonsilent mutations, with colors representing the type of functional consequences (e.g., missense or nonsense mutations; see the indicator). In the case of missense mutations, the changes in amino acid residues are also indicated. Chromosomal cytobands are shown for those with gains or losses (red and green, respectively). **B.** Chromosomal heatmaps are shown for synchronous adenoma (Ad) and carcinoma (Ca) genomes. Red and green represent the chromosomal gains and losses, respectively, from snapshots of the IGV browser.

To elucidate the individual evolutionary history, phylogenetic trees for the somatic mutations were constructed for each of the four adenoma-carcinoma genome pairs (Figure 4A). Genes implicated in CRC carcinogenesis with non-silent mutations are shown along with the copy number alterations in the trunk (clonal mutations) and two branches (adenoma- and carcinoma-specific mutations). Both *APC* and *KRAS* mutations were identified as clonal for all cases except one (MSS1). This observation supports the conventional view that *APC* and *KRAS* mutations are early events in CRC development [5]. All of the *APC* mutations were truncating (frameshifting indels or nonsense mutations), which were often accompanied by focal copy number deletions of 5q22 (MSS2 and MSS3), ensuring the biallelic inactivation of *APC* in CRC [12] (Figure 4B). All of the *KRAS* mutations were recurrent at known hotspots (e.g., G12D, G12S, and G12V). Additionally, chromosome 12, including *KRAS*, was amplified in three cases of *KRAS* mutation (MSS2, MSS3, and MSS4). In contrast to the three cases with clonal *APC* and *KRAS* mutations, one case (MSS1) exhibited a clonal *ERBB2* amplification associated with chromothripsis of 17q12 (Supplementary Figure S4). The clonal nature indicates that chromothripsis can be an early event in the CRC genome evolution [13]. Both *APC* and *TP53* mutations as well as a majority of the chromosomal copy number changes were identified as carcinoma-specific events, in this case. In addition, this case showed carcinoma-specific amplification of 7p (*EGFR* and *BRAF*) and 20q (*ASXL1, AURKA*, and *GNAS*), which were observed as clonal amplification for the three MSS cases without *ERBB2* amplification.

### Mutational map of microsatellite-unstable CRC genomes

**Figure 5:**
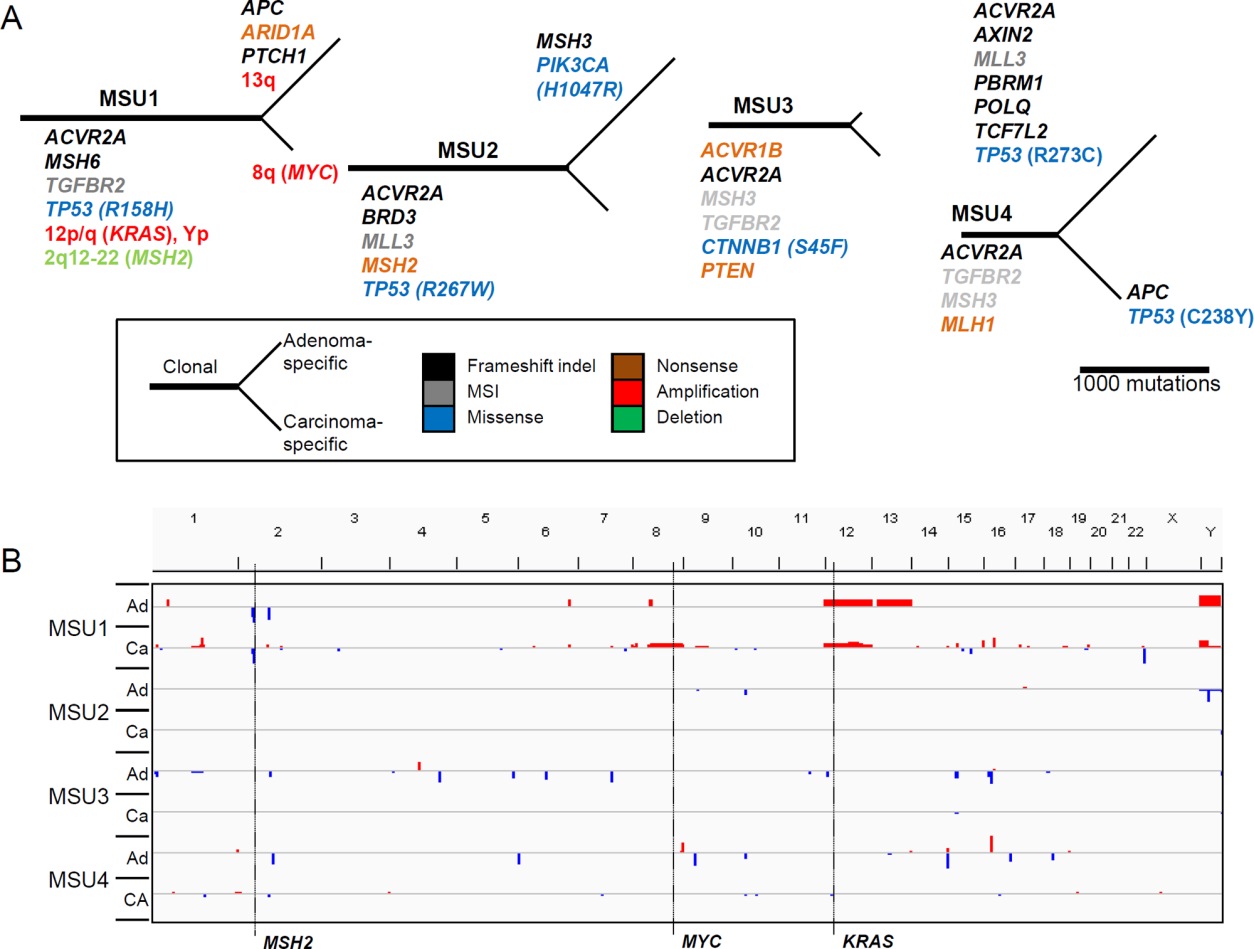
Mutational map and copy number heatmap of MSU genomes **A.** The evolutionary mutational branches are shown for four MSU cases. **B.** Chromosomal heatmaps are illustrated largely showing a deficit of copy number alterations in MSU genomes.

Figure 5 depicts the phylogenetic trees of somatic mutations and copy number changes for the four MSU adenoma-carcinoma pairs. The MSU cases were characterized by the absence of copy number changes except for a single case (MSU1) that harbored clonal focal deletion of 2q12-22 along with amplifications of 12p/q (clonal) and 8q (carcinoma-specific) encompassing *MSH2*, *KRAS*, and *MYC*, respectively. All of the four MSU cases showed ‘clonal’ truncating mutations in *ACVR2A* (frameshifting indels) and the DNA mismatch repair (MMR) genes *MSH6* (frameshifting indel in MSU1), *MSH2* (nonsense mutation in MSU2), *MSH3* (MSI events in MSU3 and MSU4), and *MLH1* (nonsense mutation in MSU4). In all cases other than for MSU2, MSI events in *TGFBR2* were recurrently observed. Our findings are not only consistent with a previous notion that MMR mutations represent early events in CRC carcinogenesis, but also suggest that perturbation of TGFβ signaling through truncating mutations in *ACVR2A* and *TGBFR2* might be early events during this process. As for Wnt/β-catenin signaling, one case harbored a clonal *CTNNB1* mutation (MSU3), but the other three potential mutations (two *APC* and one *AXIN2* frameshifting indel) in this signaling pathway were observed as lesion-specific events. In addition, frequent mutations in epigenetic modifiers (truncating mutations in *ARID1A*, *MLL3*, and *PBRM1*) were observed as clonal or adenoma-specific events.

### Class-specific mutations

To identify novel biomarker genes associated with CRC carcinogenesis, we first selected genes with preferential enrichment of nonsilent-mutations for the clonal or lesion-specific mutation categories. For the MSS cases, mutations in both *APC* and *KRAS* were relatively specific to the clonal category (*P* = 0.018 and 0.067, respectively; Fisher's exact tests) along with those of *MAP2K3* and *PCLO* (*P* = 0.09 for both). *MAP2K3* encodes MKK3, which activates p38 MAP kinase leading to phosphorylation of a number of transcription factors affecting the cell cycle [14]. The recurrent nature of the *PCLO* mutations, which was described in a recent report (35% in lymphomas), implicates the potential oncogenic roles of this gene [15]. For the adenoma-specific mutations, we identified two genes (*NPIPB5* and *MUC2*) with a substantial enrichment (*P* = 0.018 and 0.09, respectively). To cope with the hypermutability of MSU genomes, we selected genes with ≥3 truncating events. Such analyses identified three genes (*ACVR2A*, *TGFBR2*, and *SLC22A9*) and an additional two genes (*TCERG1* and *TRIM59*) whose mutations were enriched for clonal and adenoma-specific categories, respectively. For the carcinoma-specific category, there was no enriched mutation in the MSU genomes.

## DISCUSSION

Synchronous adenoma and carcinoma lesions have been analyzed in order to identify genetic alterations that are associated with malignant transformation and for tracing genomic evolution [16]. However, to the best of our knowledge, our study is the first whole exome-wide study performed on regionally synchronous colorectal adenoma-carcinoma pairs. Our evolutionary dissection of somatic mutations revealed several insights that were previously unknown. First, our mutation-based inference of clonal architectures indicated that the synchronous adenoma-carcinoma pairs might have the same clonal origin, but with independent evolutionary histories in MSU CRC. In this parallel evolutionary model (Figure 1C), a founder cell evolves into the last common ancestor, subsequently acquiring a substantial number of somatic mutations. This last common ancestor gives rise to a number of subclones, some of which will be subject to malignant transformation while others will remain in premalignant stages. This observation is notable since the parallel model is not consistent with the commonly accepted stepwise or linear progression model [5]. According to the recently suggested Big-Bang model, a single expansion in the early proliferative stages gives rise to numerous intermixed subclones that will shape the subclonal architecture and intratumoral heterogeneity of a CRC mass, in the absence of clonal sweep [17]. Our results obtained from synchronous adenoma-carcinoma pairs suggest that the divergence of adenoma-carcinoma pairs or the emergence of histologically distinct subclones may have occurred in early tumorigenesis. Consistent with the Big-Bang model, the early divergence of histologically distinct subclones may temporally coincide with the appearance of genetically distinct subclones leading to intratumoral heterogeneity. It should also be pointed out that the subclonal architecture of synchronous adenomas might not fully represent those of ‘preexisting adenomas’, since it is possible that the majority of the preceding adenomas could have been destroyed or the remaining adenomas could have undergone additional rounds of clonal sweeps. Although it has been proposed that clonal sweeps are relatively rarer than previously anticipated [17], additional lines of evidence are required to confirm whether the clonal origins of adenoma-carcinoma are unique to the synchronous cases or can be generalized to other cancer models. The evolutionary insights of early CRC carcinogenesis might also provide clues for understanding the origins of distant metastases, since it has been argued that metastases might arise from early disseminated tumors with parallel evolution independent of primary tumors [18].

In addition, an apparent advantage of comparing the mutations from synchronous premalignant and malignant lesions over the investigation of genomic snapshots derived solely from fully-developed malignant lesions might be that we can distinguish the early (clonal) mutations before the divergence of premalignant and malignant lesions from lesion-specific mutations that must have occurred after the clonal divergence. Traditionally, the loss of 5q containing *APC* has been appreciated as an initial genomic alteration in CRC [5], which is consistent with our results. Of interest, one MSS genome showed *ERBB2* focal amplification in the chromothripsis region. In this case, the majority of the somatic alterations, including *APC* and *TP53* mutations as well as copy number changes, were observed as carcinoma-specific. Such an early *ERBB2* amplification may have enabled the initiation of tumorigenesis, and the inactivation of *APC* and *TP53* followed malignant transformation, suggesting that the role of the Wnt/β-catenin pathway in *APC* loss as well as the sequence of the other genomic events in CRC is largely dependent on the mutational contexts. Also, in all of the MSU cases, truncating mutations in *ACVR2A*, *TGFBR2*, and DNA MMR genes were clonal, while *APC*, *TP53*, and *PIK3CA* mutations were frequently observed as lesion-specific alterations. These results indicate that MSU genomes may disrupt TGFβ signaling and DNA MMR as early events, followed by inactivation of other signaling pathways.

Our data suggest that an investigation of lesion-specific alterations might provide additional insight into CRC carcinogenesis. We observed that indels were largely lesion-specific, rather than clonal, in both MSS and MSU genomes. Given that frameshifting indels within coding regions are often deleterious, it is possible that during malignant transformation, the genomes might be more permissive to deleterious events or more tolerable to mutational burdens than those during initial cellular proliferation. Moreover, we did not observe any recurrent carcinoma-specific mutational events in the CRCs, further suggesting that many of the lesion-specific mutations (including indels and MSI events) represent those not yet selected and fixed, rather than those that are functionally selected. It is plausible that in CRC, the genetic makeup for malignant potential is achieved earlier, before the divergence of malignant clones from the common ancestor, while the later events are rather stochastic. This hypothesis is consistent with a previous assumption that the accumulation of genomic events is more important than the sequence of the events [5], but requires further investigation in a larger cohort.

In this study, we explored adenoma-carcinoma pairs from eight CRCs (four MSU and four MSS genome pairs), the number of which analyzed might be a limitation for the relevance of this study. As we used only histologically defined, synchronous adenoma-carcinoma cases in frozen state, it was very difficult to enroll more samples in this study. It will be possible to analyze a larger cohort with formalin-fixed and paraffin-embedded tissues by using a targeted next-generation sequencing approach. Further investigation with a larger CRC cohort from multi-ethnic groups will be required to validate whether the parallel evolution model of synchronous adenoma-to-carcinoma is a universal phenomenon. Exploration of recurrent alterations in the branches of carcinoma-specific mutations with distinct mutational contexts such as *ERBB2* amplification preceding *APC* and *KRAS* mutations would also be valuable information for understanding the evolutionary process of CRC.

In summary, our study analyzing synchronous colorectal adenoma-carcinoma pairs found that a ‘parallel’ rather than traditional ‘stepwise’ model might represent CRC evolution, in many cases. We report a case in which a focal *ERBB2* amplification might have arisen before *APC* and *TP53* mutations. This case suggests that the mutational consequences can be context-dependent and can be the source of intertumoral variability of the CRC mutational landscape. Moreover, *ERBB2* amplification represents an optimal candidate of an addicted, actionable item with available inhibitors (*ERBB2* antibody, trastuzumab) [19] highlighting that mutational architecture analysis might have clinical applications.

## MATERIALS AND METHODS

### Tumor specimen

Colectomy tissues from eight CRC patients used for this study came from a university-affiliated hospital (Eujeongbu St. Mary Hospital, Korea). All of the patients were Koreans and we only collected sporadic cases without any positive family history of CRC. Approval for this study was obtained from the Catholic University of Korea, College of Medicine's institutional review board. Clinicopathologic features of the right CRC patients were summarized in Table 1. By examining frozen sections, we identified adenoma tissues attached to invasive carcinomas (Figure 1A). Next, the frozen tissues with adenomas associated with carcinomas were serially cut and lightly stained with hematoxylin without any fixation. Adenoma cells and carcinoma cells were selectively procured from hematoxylin-stained frozen sections using a 30G1/2 hypodermic needle by microdissection as described previously [20]. Adenoma and carcinoma cell purities of the microdissection were approximately 75%–80%. To minimize DNA degradation, we finished the processes from cutting to microdissection within 120 min. For normal DNA, we used frozen tissue blocks that were devoid of adenoma and carcinoma cells. For genomic DNA extraction, we used the DNeasy Blood & Tissue Kit (Qiagen, Hilden, Germany) according to the manufacturer's recommendation.

### Sequencing and somatic variants

WES was performed for the genomic DNA obtained from tumor and matched normal specimen using Agilent SureSelect Human All Exome 50 Mb kit (Agilent Technologies) and Illumina HiSeq2000 platform. The acquisition and processing of the sequencing data was performed as previously described [21]. The preparation of genomic DNA libraries and the generation of 101bp paired-end sequencing reads were performed according to the manufacturer's instructions. General information including the sequencing depth and target coverage is shown in Supplementary Table S2. We first used Burrows-Wheeler aligner (BWA) [22] to align the paired-end sequences onto the UCSC hg19 human reference genomes. Local alignments and the score recalibration of the sequencing reads were performed using the Genome Analysis ToolKit [23]. For additional processing and the management of the sequencing data were done using Picard (http://picard.sourceforge.net) and Samtools [24]. We used Mutect [25] and SomaticIndelDetector [23] to call the somatic single nucleotide variants and small indels by comparing the sequencing reads from the adenoma and carcinoma genomes with those from the matched normals. ANNOVAR package was used to intersect the mutations on coding sequences and also to annotate the functional consequences of somatic variants [26].

### Microsatellite instability

The MSI events were identified as previously described [27]. First, we collected mRNA sequences of 39,496 RefSeq genes from UCSC genome browser (http://genome.ucsc.edu/). Then, we used Sputnik (http://espressosoftware.com/sputnik/) to identify microsatellite repeats in the RefSeq sequences. We limit the unit length (mono-, di-, tri-, and tetra-nucleotide repeats) and the size of the repeats (7 to 60 bp) identifying a total of 146,447 ‘reference’ microsatellite repeats in RefSeq sequences. Second, we obtained the repeat length distribution for each of the reference microsatellite repeats by collecting the lengths of all intraread microsatellite repeats that are mapped to the corresponding locus. Then, the statistical difference in the length distribution was estimated by Kolmogorov-Smirnov tests for genome pairs to be analyzed (e.g., carcinoma-*vs*-normal or adenoma-*vs*-normal genomes) per case. Significant (false discovery rates or FDR <0.05) difference was considered as an MSI event. A total of 339 MSI events were identified. About 40% of MSI events (135 out of total 339 MSI events) regionally coincided with indel calls. Given the higher sensitivity of the MSI calling algorithms [28], it is likely that those indels represent clonally fixed MSI events; thus, we include 204 MSI events that do not overlap with any indels in the final list of 11,250 genomic variants (Supplementary Table S1).

### Copy number inference

For copy number profiling, we used VarScan2 [29] to obtain the read depth differences between the tumor and matched normal exome sequencing data. The GC-corrected read depth was log2-transformed and segmented using circular binary segmentation algorithm [30].

### Clonal analyses of mutation

For unsupervised clustering of mutations, we used dbscan algorithm as previously described [10]. To avoid the need for copy number correction, the somatic mutations residing in the copy number neutral genomic segments were used for MSU1. For the three remaining MSU genomes (MSU2, MSU3 and MSU4) without apparent copy number changes, we collected all the somatic mutations in autosomes for clonal analyses. The clustering was done as previously described [10]. Outliers were identified using dbscan using a reachability distance of 1%. The clusters containing less than 1% of total number of mutations were ignored as outlier mutations.

## SUPPLEMENTARY FIGURES AND TABLES




