# Supplementary material for: Clonal origins and parallel evolution of regionally synchronous colorectal adenoma and carcinoma
Source: Oncotarget. 2015 Aug 7;6(29):27725–35. doi: 10.18632/oncotarget.4834 (PMC4695021; doi:10.18632/oncotarget.4834)
Supplement: Supplementary file 1 [file oncotarget-06-27725-s001.pdf]

SUPPLEMENTARY FIGURES AND TABLES

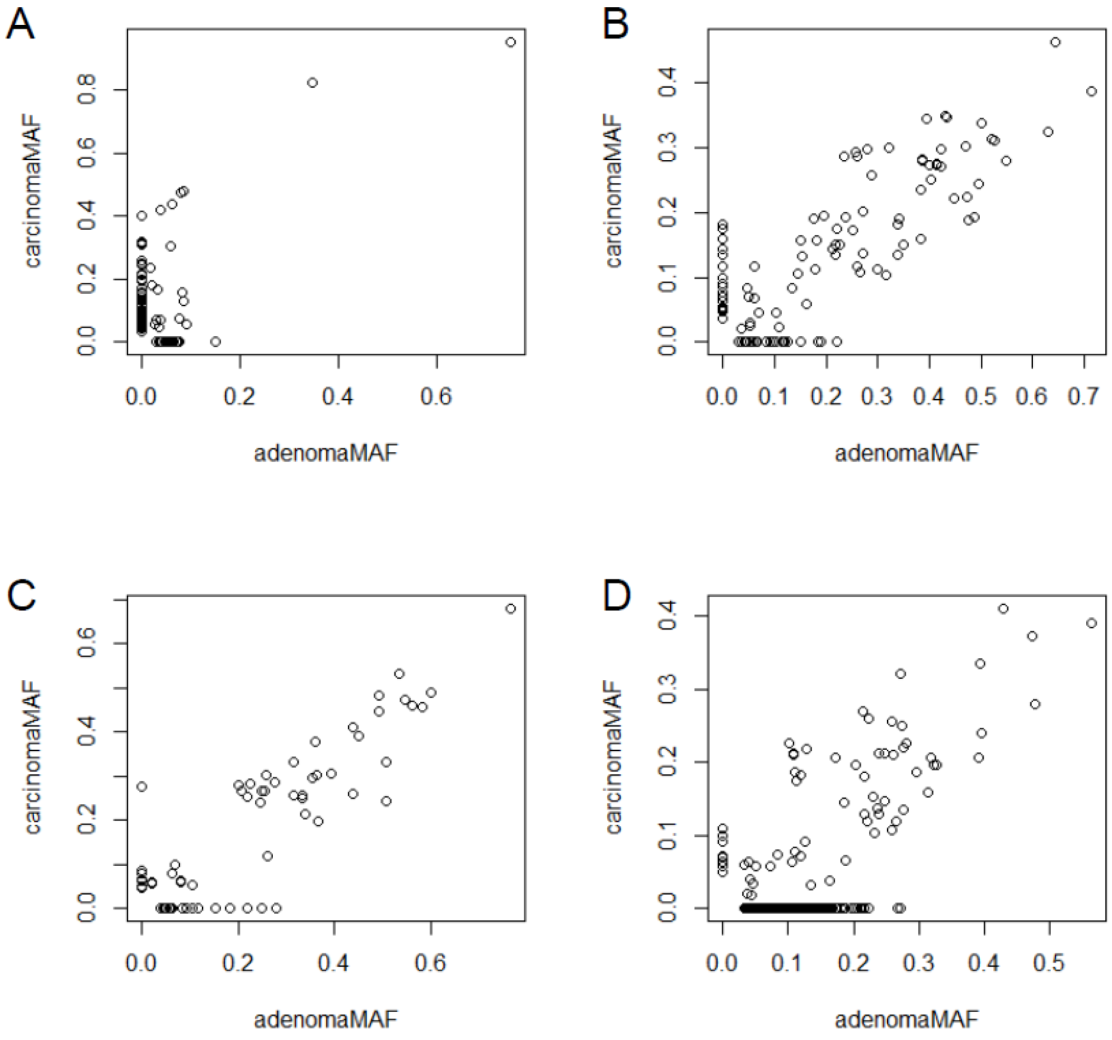

Supplementary Figure S1: Clonal analyses using mutational abundance in regionally synchronous colorectal lesions in MSS genomes. A–D. MAF plots of MSS1-4 genomes.

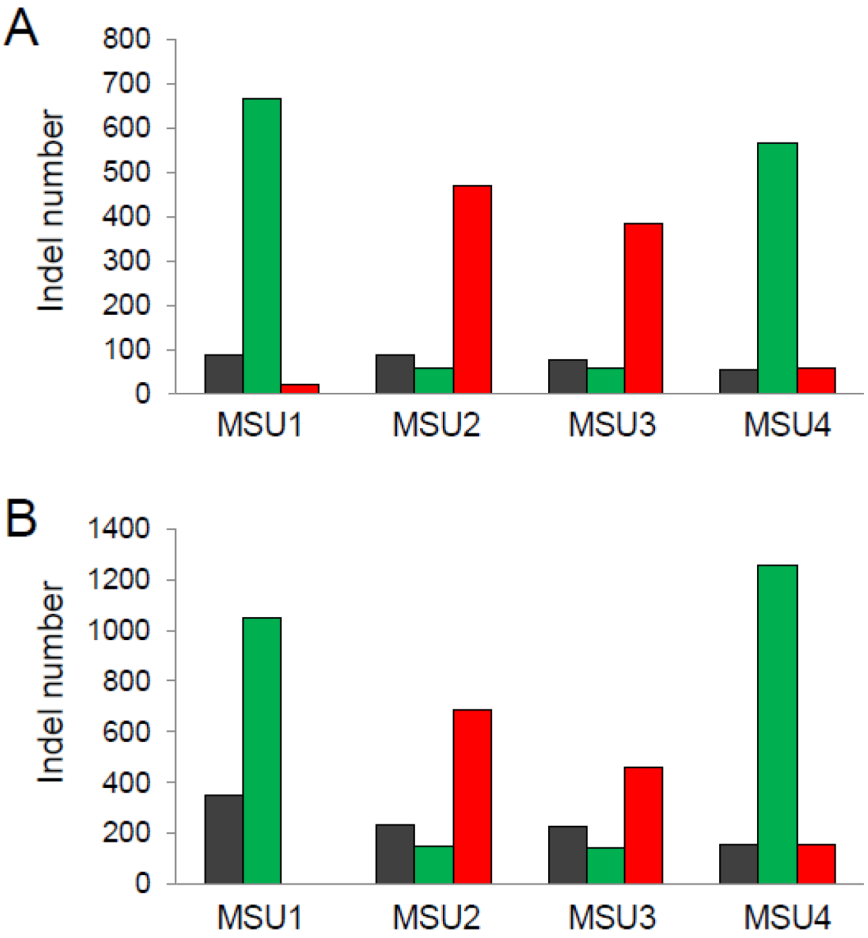

Supplementary Figure S2: The abundance of high-confident indels. A. For four microsatellite-unstable genomes, the number of indels that were identified in the regions > X50 are shown. **B.** The indels whose MAF > 0.4 are shown.

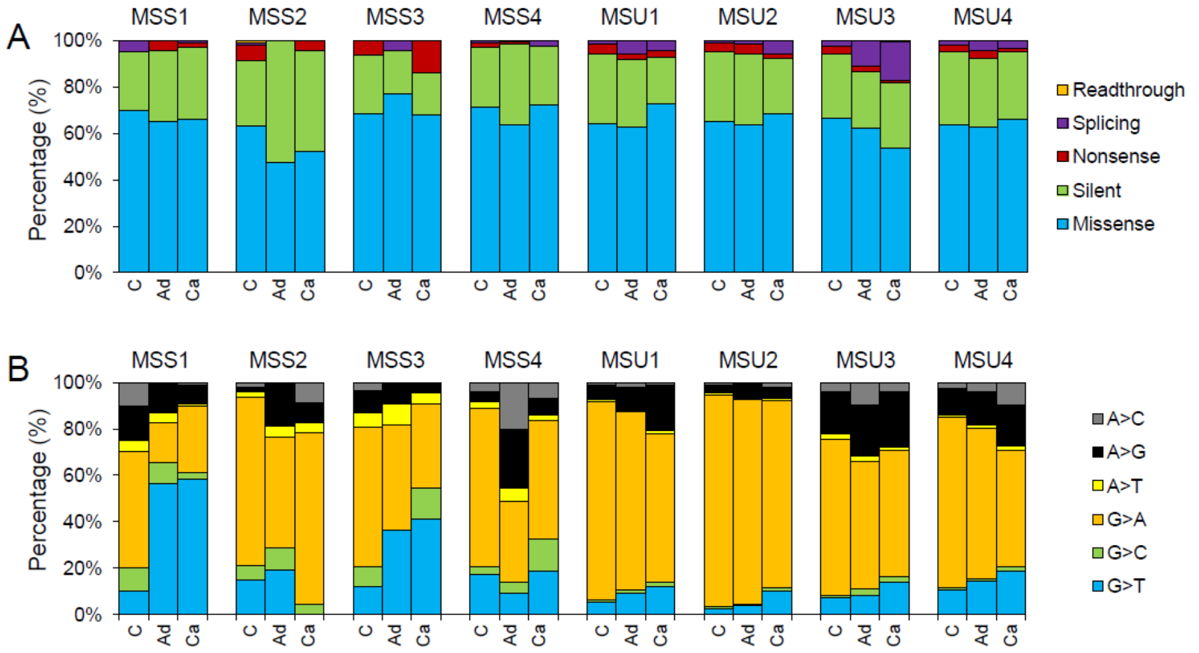

Supplementary Figure S3: Relative fraction of SNV. A. The percentage (%) of functional annotations of SNVs are shown. For each case, Clonal (C) and adenoma-/carcinoma-specific (Ad and Ca, respectively) mutations are separately shown. **B.** The six mutational spectra is shown similarly.

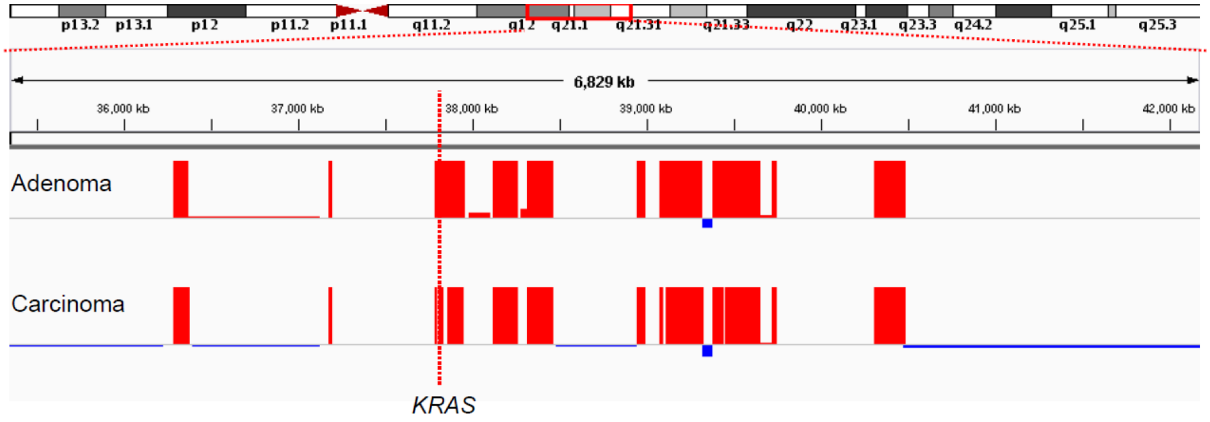

Supplementary Figure S4: Chromothripsis event associated with ERBB2 amplification. The copy number profiles of MSS1 is shown (above and below for the adenoma and carcinoma, respectively). The fragmented amplifications (red) are the evidence of chromothripsis. The KRAS locus is indicated with a dotted red line.

Supplementary Table S1: Somatic variants identified across eight synchronous pairs of colorectal high-grade adenomas and carcinomas

Supplementary Table S2: The description of whole-exome sequencing data

Samples*	Sequencing reads**	Mapped (%)	Coverage (mean)	Coverage (median)	% of bases (>= 20 reads)
MSS1 (A)	57,784,167	56,345,144 (97.5%)	75.23	58	87.9%
MSS1 (N)	49,020,155	47,911,643 (97.7%)	62.32	50	86.8%
MSS1 (T)	49,557,224	48,228,107 (97.3%)	63.01	47	85.1%
MSS2 (A)	59,340,417	58,127,361 (98.0%)	77.71	59	88.0%
MSS2 (N)	54,066,095	53,262,471 (98.5%)	67.93	52	86.1%
MSS2 (T)	61,682,598	60,780,464 (98.5%)	76.98	59	87.7%
MSS3 (A)	55,793,629	55,069,829 (98.7%)	70.75	53	86.5%
MSS3 (N)	64,126,205	63,217,888 (98.6%)	80.11	62	87.7%
MSS3 (T)	50,531,627	49,870,718 (98.7%)	65.74	49	85.9%
MSS4 (A)	60,264,017	59,003,103 (97.9%)	79.12	61	88.4%
MSS4 (N)	53,478,185	52,704,181 (98.6%)	67.1	52	86.0%
MSS4 (T)	52,982,350	51,814,748 (97.8%)	66.81	51	86.0%
MSU1 (A)	63,672,071	62,276,937 (97.8%)	84.43	65	88.6%
MSU1 (N)	57,072,682	55,699,333 (97.6%)	75.65	59	88.3%
MSU1 (T)	39,002,391	38,679,069 (99.2%)	48.2	36	77.7%
MSU2 (A)	54,997,283	53,772,725 (97.8%)	71.4	55	87.4%
MSU2 (N)	38,637,740	38,302,285 (99.1%)	48	36	77.8%
MSU2 (T)	59,004,527	58,153,461 (98.6%)	73.71	56	87.6%
MSU3 (A)	49,425,859	46,938,027 (95.0%)	63.91	49	85.5%
MSU3 (N)	39,747,075	39,398,455 (99.1%)	48.42	36	78.0%
MSU3 (T)	59,592,919	58,037,982 (97.4%)	76.71	60	88.4%
MSU4 (A)	57,031,271	56,226,677 (98.6%)	72.77	56	87.9%
MSU4 (N)	61,520,152	60,650,785 (98.6%)	79.71	61	88.4%
MSU4 (T)	58,162,505	55,904,859 (96.1%)	71.61	55	85.8%

*The synchronous adenoma (A) and tumor (T) are discriminated with matched normal genome (N). Eight cases are annotated according to the status of microsatellite instability, e.g., four microsatellite stable (MSS1-4) and four microsatellite-unstable genomes (MSU1-4).

**The mean and median coverage as well as the % of bases (>= 20 reads) were calculated onto the targeted regions (Agilent SureSelect 50 Mb exon).
